# Pentobarbital Induced Hypokalemia: A Worrying Sequela

**DOI:** 10.1016/j.ijscr.2020.05.032

**Published:** 2020-05-29

**Authors:** Mark Awad, Joyce Bonitz, Abimbola Pratt

**Affiliations:** Department of Surgery, Hackensack Meridian Jersey Shore University Medical Center, 1945 NJ-33, Neptune City, NJ, 07753, United States

**Keywords:** Barbiturate coma, Intracranial hypertension, Traumatic brain injury, Pentobarbital, Thiopental, Hypokalemia

## Abstract

**Introduction:**

Intracranial hypertension that is not responsive to other therapies can be managed through the use of a barbiturate induced coma. Although potentially effective, there are known complications associated with this treatment, and as such it is typically reserved for the most severe cases. One such sequela of barbiturate induced coma therapy is refractory hypokalemia and subsequent rebound hyperkalemia.

**Presentation of case:**

This case report discusses a patient who experienced hypokalemia during pentobarbital induced coma for unmanageable elevations in intracranial pressure and was treated conservatively to avoid rebound hyperkalemia depicting successful deployment of permissive hypokalemia.

**Discussion:**

It is vital that clinicians understand the possible adverse effects associated with barbiturate induced coma therapy, and that a careful balance be struck between hypokalemia and potassium supplementation to avoid rebound hyperkalemia.

**Conclusion:**

Given that the risk of rebound hyperkalemia is of significant concern in patients who experience hypokalemia on barbiturate induced coma therapy, permissive hypokalemia can be a viable treatment option achieved by lowering the potassium replacement target threshold in such patients.

## Highlights

• High dose barbiturate use has been linked to life threatening hypokalemia and rebound hyperkalemia.
• Pentobarbital is a barbiturates that is used in high doses to induce a coma state to lower intracranial pressures.
• Through constant monitoring of potassium levels and permissive hypokalemia, avoidance of rebound hyperkalemia is achievable.

## 1 Introduction

High-dose Barbiturates, and in particular pentobarbital have been effective in controlling intracranial pressure (ICP) in patients that have undergone standard medical and surgical therapies without success [1]. Typical initial therapy for an elevated ICP should include standard therapies such as mannitol, hypertonic saline, normothermia maintenance, hyperventilation, and elevation of head to a 30-degree angle in order to maintain a therapeutic target of <20 mmHg ICP and >60 mmHg cerebral perfusion pressure (CPP) [2]. Should the standard therapies fail, high-dose barbiturates can be used as a second line approach to effectively manage ICP via several mechanisms including alterations in vascular tone, suppression of metabolism, and inhibition of free radical mediated lipid peroxidation [3]. The majority of current publications examine the use of thiopental, including its effectiveness at reducing ICP as well as adverse effects associated with it. One such study conducted reported that the vast majority of its participants experienced complications associated with thiopental use. The study reported 87 % of participants had hepatic dysfunction, 82 % had hypokalemia, 76 % had respiratory complications (8 % of whom developed acute respiratory distress syndrome), 55 % had infections, with the majority due to *Staphylococcus aureus* lower respiratory tract infections, 58 % had arterial hypotension, and 47 % had renal dysfunction [4]. One of the most potentially life threatening of these complications is the development of severe refractory hypokalemia and a rebound hyperkalemia due to excessive repletion. This case presentation shows that with proper monitoring and management, the rebound hyperkalemia may be nullified. This work has been reported in line with SCARE criteria [5].

## 2 Case presentation

**Fig. 1:**
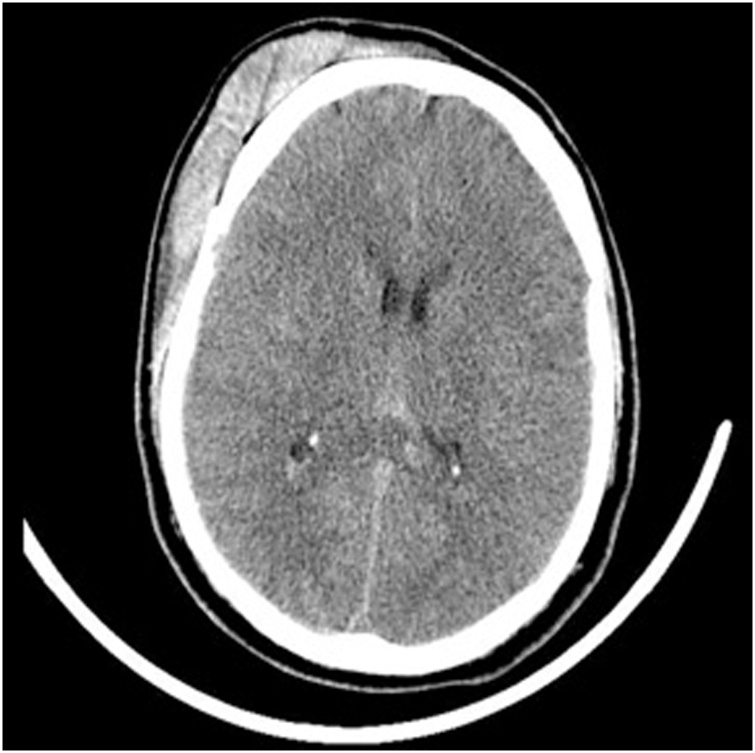
CT scan of head on admission showing subdural, subarachnoid, intraperenchymal hemorrhages and small areas of brainstem hemorrhage.

A 36-year-old male pedestrian with no past medical history according to family who was at bedside was struck by a vehicle which was reported by witnesses to be going approximately 50mph. The man was reported to have been found by emergency personnel approximately 75 feet from the impact site. He was intubated on the scene and presented to the trauma bay with a Glasgow coma scale (GCS) of 3 T. Computed tomography (CT) of the brain showed evidence of traumatic brain injury including subdural, subarachnoid, intraparenchymal hemorrhages, and small areas of hemorrhage in brainstem consistent with diffuse axonal injury (Fig. 1).

Neurosurgery was immediately consulted and recommended no surgical intervention at that time, the patient be placed on mannitol for presumed increases in ICP, Levetiracetam for seizure prophylaxis, continued ventilator for breathing, and that a repeat head CT be done in 6 h.

The next morning (Day 1), a repeat head CT showed global cerebral edema and a new right frontal epidural hematoma. At that time the patient met criteria for invasive ICP monitoring and was taken to the operating room for device placement through a left frontal burr hole. The ICP monitor was placed in the left frontal lobe with an opening pressure of 35 mmHg and within minutes of elevation of the head of the bed, hyperventilation, and hypertonic saline infusion had lowered to 10 mmHg (goal <20 mmHg). The patient was then transferred back to the surgical intensive care unit for monitoring and care where his hypertonic saline was continued with a goal sodium of 145−155. His Propofol was continued for pain control, and the patient was placed on norepinephrine, phenylephrine, and vasopressin to maintain a CPP between 50−70 mmHg, and mean arterial pressures (MAP) >65.

**Fig. 2:**
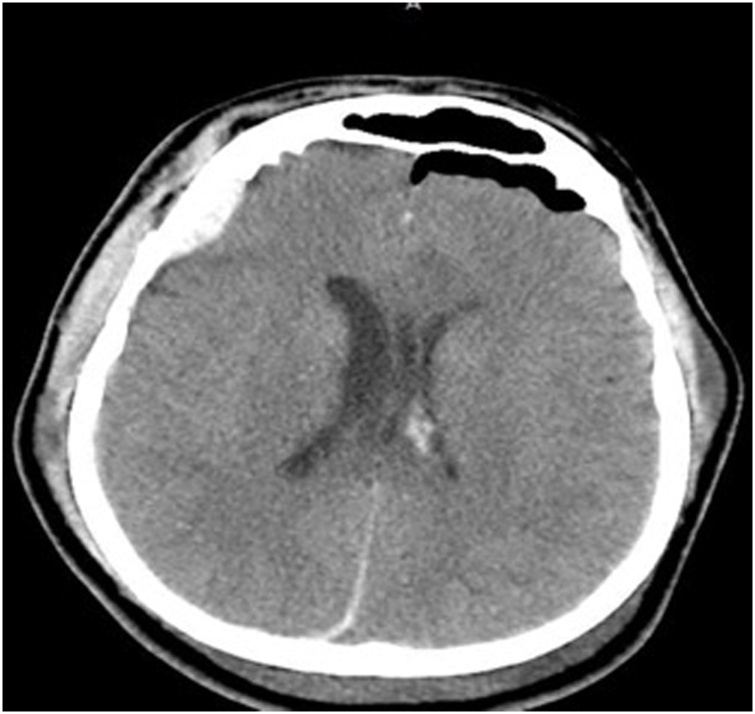
CT scan of head on day 3 showing progressive ventricular widening and frontal pneumocephalus.

On day 2, a repeat head CT showed no increase in intra or extra axial collections, the vasopressors started to be weaned, the patient had a tracheostomy placed percutaneously for breathing via ventilator, and his potassium was measured to be 4.4. The next day, the patient was continued on norepinephrine and vasopressin to maintain goal pressures and underwent a repeat head CT which showed progressive ventricular widening and frontal pneumocephalus (Fig. 2). The patient was given a bolus of pentobarbital (10 mg/kg over 30 min followed by 15 mg/kg over 3 h) with a continuous infusion started at 1 mg/kg titrated up to 3 mg/kg to achieve burst suppression and placed on a video EEG to monitor brain activity during his induced coma. His ICP monitor was also adjusted by the neurosurgery team due to a kink in the fiberoptic cable causing fluctuating ICP readings ranging from -4 to 50 mmHg, after adjustment the ICP was reported to be steadily reading 50 mmHg and the patient was tachycardic and hypertensive.

On day 4, his potassium 10 h after pentobarbital infusion was started measured 3.6, the patient had an ICP of 22 with EEG showing burst suppression, and the neurosurgery team recommended keeping the patient in a pentobarbital induced coma for 5–7 days through the maximal swelling period and slowly titrating it down after that. 12 h after the last measurement of potassium, a repeat study showed a potassium concentration of 1.6 mmol/L and the patient was given 8 doses of 20 mEq/h of potassium chloride.

**Graph 1:**
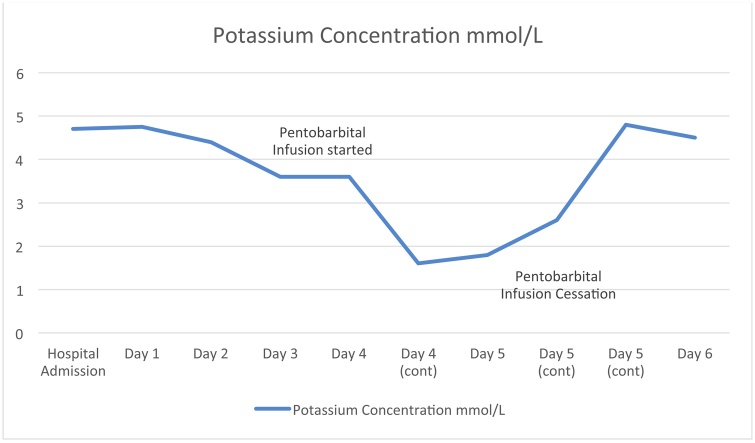
Graphical depiction of potassium concentration in relation to Pentobarbital infusion.

The next day, 4 h after the previous potassium measurement, potassium was measured to be 1.6 and after potassium chloride supplementation, serum potassium only increased to 1.8. Due to fear of arrhythmia the pentobarbital was titrated down over a period of 12 h and replaced by increasingly high dose Midazolam continuous infusion starting at 15 mg/hr and achieving burst suppression at 50 mg/hr. The patient’s potassium concentration 12 h after stopping the pentobarbitol infusion was 4.8 and has remained between 3.6 and 4.8 for the remainder of his stay in the SICU (Table 1 and Graph 1 ). The patient was stable hereafter and later transitioned to propofol and fentanyl instead of the midazolam and remained in the hospital with a GCS of 3 T for the total follow up period of 30 days and was later transferred to a long term acute care facility still with a GCS of 3 T, in a persistent vegetative state.

**Table 1:** Potassium concentration in relation to intracranial pressure and Pentobarbital infusion.

Day #	Time Since Pentobarbital Infusion Started	Time Since Pentobarbital Infusion Cessation	Potassium Concentration (mmol/L)	Intracranial Pressure (mmHg)
0	–	–	4.7	Unknown
1	–	–	4.6−4.9	10−35
2	–	–	4.4	15
3	2 h	–	3.6	50
4	10 h	–	3.6	22
4	22 h	–	1.6	22
5	26 h	Cessation Begun	1.8	15−20
5	30 h	4 h	2.6	15−20
5	38 h	12 h	4.8	15−20
6	50 h	24 h	4.5	15−20

## 3 Discussion

High-dose barbiturate induced coma therapy is known to be an effective method in controlling elevated intracranial pressures that are refractory to medical and surgical treatments. A review of barbiturate coma therapy (BCT) in 92 semicomatose patients with a Glasgow Coma Scale (GCS) of 7 or less with elevated ICP refractory to typical therapy reported better outcomes of patients that underwent BCT therapy compared to a control group which did not receive BCT. At 3 months after insult a Glasgow outcome scale (GOS) was reported and patients with BCT therapy had a good outcome, defined as a GOS of 4 or 5, 27.5 % of the time compared with 5.8 % in the control group (p < .01) [6]. Although, the use of BCT is indicated and useful in patients who experience an elevated ICP the threat of severe and life threatening dyskalemia must be kept in mind during administration of high dose barbiturates. Potassium monitoring is vital in the setting of high-dose barbiturate use in order to ensure that hypokalemia is recognized early and treated effectively while also not overtreating it to avoid rebound hyperkalemia. The leading hypothesized mechanism for BCT induced hypokalemia and subsequent hyperkalemia revolves around neuronal voltage-dependent potassium currents. The authors of leading research in this field found that barbiturates reversibly inhibit neuronal voltage-dependent potassium currents which leads to a decrease in extracellular potassium and increase of intracellular potassium in a dose dependent manner [7]. This may then be reversed upon cessation of barbiturate therapy and subsequently lead to increased extracellular potassium if there has been rapid administration of potassium supplementation. In a more recent case series examining the relationship between BCT and electrolyte abnormalities, the mean potassium replacement during hypokalemia was higher in patients who developed hyperkalemia compared to those who did not 230 ± 135 mmol versus 66 ± 70 mmol) [8]. The report suggested that a lower potassium replacement target threshold of 3.0 mmol/L might be appropriate in the absence of cardiac arrhythmias. Current research is emerging that states other options for coma therapy may be as effective for intracranial pressure control with lower risks of dyskalemia. In one such study which analyzed 60 patients who received coma therapy for increased intracranial pressures using either propofol or thiopental, propofol was less frequently associated with moderate to severe hypokalemia (K < 3.0 mmol/L) in comparison to thiopental (13 % vs. 51.4 %, p = 0.003) and less frequently associated with rebound hyperkalemia (K > 5.0 mmol/L) following cessation of infusion (8−4.3% vs 32.4 %, p = 0.010) [9]. With careful monitoring of potassium concentrations, potassium supplementation, titration of barbiturates, and use of other analgesics to achieve the desired coma therapy, patients stand to benefit in both short term and long term outcomes.

## 4 Conclusion

In anticipation of both hypokalemia and hyperkalemia this patient’s potassium level was monitored often and repeatedly. Although the patient did develop hypokalemia, he did not develop hyperkalemia. This is owed to both careful use of potassium repletion, ensuring that upon cessation the patient was not inundated with extracellular potassium, and a gradual titration of the pentobarbital infusion in order to closely monitor potassium levels. Although further research is needed examining the safety of limiting potassium repletion and lowering the potassium target, this case presentation has demonstrated that it is an effective deterrent of rebound hyperkalemia. It is therefore recommended that clinicians are not only aware of this sequela associated with high dose barbiturate use, but actively monitor potassium before, during, and after barbiturate infusion. It is also recommended that potassium repletion should not exceed a target threshold of 3.0 mmol/L in the absence of cardiac arrhythmias. Future studies are needed to evaluate other means of correcting hypokalemia that will not result in hyperkalemia upon barbiturate cessation.

## Declaration of Competing Interest

Nothing to declare.

## Funding

Nothing to declare.

## Ethical approval

NA.

## Consent

Informed consent was obtained for publication of this case report and accompanying images. A copy of the written consent is available for review by the Editor-in-Chief of this journal on request.

## Author contribution

Mark Awad-Data collection and analysis, formulation of manuscript.

Joyce Bonitz-Study concept.

Abimbola Pratt- Study design.

## Registration of research studies

1 Name of the registry: NA.
2 Unique identifying number or registration ID: NA.
3 Hyperlink to your specific registration (must be publicly accessible and will be checked): NA.

## Guarantor

Mark Awad, Joyce Bontiz, Abimbola Pratt.

## Provenance and peer review

Not commissioned, externally peer-reviewed.
